# Comprehensive Development and Implementation of Good Laboratory Practice for NGS Based Targeted Panel on Solid Tumor FFPE Tissues in Diagnostics

**DOI:** 10.3390/diagnostics12051291

**Published:** 2022-05-23

**Authors:** Anuradha Chougule, Vinita Jagtap, Ankita Nikam, Shrutikaa Kale, Kavya Nambiar, Priyanka Bagayatkar, Pratik Chandrani, Rajiv Kaushal, Vanita Noronha, Vijay Patil, Shripad Banavali, Kumar Prabhash

**Affiliations:** 1 Medical Oncology-Molecular Laboratory, Tata Memorial Hospital, Ernest Borges Marg, Parel, Mumbai 400012, Maharashtra, India; vinbiotech4u@gmail.com (V.J.); nikam.ankita04@gmail.com (A.N.); shrutikaa.kale@gmail.com (S.K.); kavyanambiar396@gmail.com (K.N.); priyanka.baga080@gmail.com (P.B.); pratikchandrani@gmail.com (P.C.); 2 Homi Bhabha National Institute (HBNI), Training School Complex, Anushakti Nagar, Mumbai 400094, Maharashtra, India; rajiv.kaushal@gmail.com (R.K.); vanita.noronha@gmail.com (V.N.); vijaypgi@gmail.com (V.P.); banavali_2000@yahoo.com (S.B.); 3 Department of Pathology, Tata Memorial Centre, Ernest Borges Marg, Parel, Mumbai 400012, Maharashtra, India; 4 Department of Medical Oncology, Tata Memorial Hospital, Mumbai 400012, Maharashtra, India

**Keywords:** standardization, targeted panel, libraries, FFPE, solid tumors, next-generation sequencing (NGS), quality check (QC), diagnostics

## Abstract

The speed, accuracy, and increasing affordability of next-generation sequencing (NGS) have revolutionized the advent of precision medicine. To date, standardized validation criteria for diagnostic accreditation do not exist due to variability across the multitude of NGS platforms and within NGS processes. In molecular diagnostics, it is necessary to ensure that the primary material of the FFPE sample has good quality and optimum quantity for the analysis, otherwise the laborious and expensive NGS test may result in unreliable information. Therefore, stringent quality control of DNA and RNA before, during, and after library preparation is an essential parameter. Considering the various challenges with the FFPE samples, we aimed to set a benchmark in QC metrics that can be utilized by molecular diagnostic laboratories for successful library preparation and high-quality NGS data output. In total, 144 DNA and 103 RNA samples of various cancer types with a maximum storage of 2 years were processed for 52 gene focus panels. During the making of DNA and RNA libraries, extensive QC check parameters were imposed at different checkpoints. The decision tree approach can be set as a benchmark for FFPE samples and as a guide to establishing a good clinical laboratory practice for targeted NGS panels.

## 1. Introduction

Advances in sequencing technology such as massively parallel sequencing, also known as next-generation sequencing (NGS), have led to a paradigm shift in oncology companion diagnostics. It has progressed from a single gene-based test to multiplexed next-generation sequencing-based assay for the identification of frequent somatic driver actionable alterations in solid tumors [1,2,3].

The speed, accuracy, and increasing affordability of next-generation sequencing (NGS) have revolutionized the advent of precision medicine, which involves custom-made treatment based on disease-driving molecular aberrations [4].

To date, standardized validation criteria for diagnostic accreditation do not exist due to variability across the multitude of NGS platforms and within NGS processes.

In routine molecular diagnostics for solid tumors, the starting material is in the form of biopsies preserved as formalin-fixed paraffin-embedded (FFPE) blocks. Adequate long-term storage of the FFPE blocks is a valuable benefit that aids in molecular diagnostics and translational cancer research. The congruence of pre-analytic procedures represents the cornerstone of optimal DNA integrity in molecular pathology [5]. In the era of precision oncology, the utility of DNA and RNA for whole-exome and targeted sequencing is increasingly popular on the next-generation sequencing (NGS) platform. Because of the huge amount of raw data analysis, whole-genome and transcriptome are still at the research level. The quality and quantity of extracted DNA and RNA from the archival biopsies differ widely due to age, fixation conditions, DNA-protein crosslinking, and reagent inhibitors, which may affect downstream genomic analyses. It is necessary to ensure that the primary material has good quality and optimum quantity for the analysis, otherwise the laborious and expensive NGS test may result in unreliable information [6]; this was shown in a study where 70% of library preparation for sequencing was unsuccessful due to bad DNA integrity [7]. Another important aspect, to date there is no established cutoff wherein the FFPE material should not be used for sequencing at the cost of calling artifacts for mutations [6]. In addition, the quantity and the condition of the nucleic acid trapped in such diverse clinical samples have proven to be significant barriers and can lead to low read depth and allele dropout [8].

Therefore, stringent quality control of DNA and RNA before, during, and after library preparation is an essential parameter; however, to date, there are no precise criteria to identify the high-risk failures in FFPE DNA samples to run on the NGS platform. Some of the literature suggests there is a strong correlation between initial DNA quality and corresponding library concentration [9]. In the context of next-generation sequencing, the old saying “garbage in, garbage out” translates to “poor sample preparation results in poor data”. Thus, apart from bioinformatics standardization, there is a need to set some critical parameters for the initial QC check while working with non-renewable FFPE-derived nucleic acid samples for NGS. The critical variables in library preparation can profoundly affect the sample quality and output of the results.

The specimen yield is often a limiting factor while studying the non-renewable FFPE clinical samples; the ability to extract and assess DNA quality with a minimal amount of material has a paramount significance as reported. A lack of integrity standards may generate inconsistent and non-reproducible results [6,10]. Tumors have a very rapid growth rate and hence the time taken to offer treatment to a cancer patient is the most crucial parameter in cancer care. To achieve confidence and reliability of reports, it is mandatory to implement the QC parameters at every step [11] while performing the NGS wet-lab procedures. Considering the various challenges with the FFPE samples, we aimed to set a benchmark in QC metrics that can be utilized by molecular diagnostic laboratories for successful library preparation and good quality NGS data output.

## 2. Materials and Methods

### 2.1. Nucleic Acid Extraction and Quality Assessment

In this study, 144 FFPE DNA and 103 FFPE RNA samples of various cancer types with a maximum storage of 2 years were included. FFPE samples with 20–30% of tumor content were accepted for the assay. Depending upon the size and site of the tissue, three curls with sections of 7–10 μm thickness were processed for extracting DNA and RNA. The conventional protocol of deparaffinization using limonene as an organic solvent was conducted. Nucleic acid extraction was performed using a QI Amp DNA FFPE and Qiagen RNeasy FFPE Kit as per the KIT protocol. Extracted nucleic acid was checked for purity by assessing 260/280 and 260/230 ratios. To measure the integrity of DNA and RNA, specific screen tapes were used on an Agilent 4200 TapeStation. The instrument by default determines the DIN and RIN values of the samples. The distribution value (DV) for RNA was calculated from the electropherogram using the TapeStation Analysis software. dsDNA and RNA quantification was performed on a Qubit fluorometer using a dsDNA BR Assay and RNA HS Assay Kit, respectively. cDNA was synthesized using AmpliSeq cDNA Synthesis for Illumina as per the protocol and RNA was taken neat for cDNA synthesis assay [12]. Furthermore, for the housekeeping genes β actin and GAPDH of 100 bp and 400 bp, respectively, a PCR amplification assay was performed on all the cDNA samples.

### 2.2. Library Preparation

Library preparation: Libraries were prepared using an AmpliSeq Illumina Focus Panel. It is an amplicon-based targeted panel with DNA and RNA pool-investigating mutations across 52 genes with known relevance to solid tumors. Libraries were prepared as per the protocol described in AmpliSeq for the Illumina Focus Panel Reference Guide. The recommended input for DNA and RNA for the library was 1–100 ng. Because of the disparity in nucleic acid concentration from each sample, based on optimization, samples were normalized with nuclease-free water to attain the final concentration of 90 ng DNA. Low-quantity DNA was taken neat.

PCR amplification of DNA and cDNA was performed. Amplicons were then partially digested and ligated to uniquely brocaded index-adaptor sequences provided by Illumina. Libraries purified with Agencort AMPure XP beads were further analyzed for quality on an Agilent 4200 TapeStation using high-sensitivity D1000 screen tape and a high-sensitivity D1000 reagent

During the library preparation, two more QC check of DNA libraries was performed by using a TapeStation to measure the presence of desired amplicons.

### 2.3. Library Normalization

All libraries were quantified using a Qubit Fluorometer using a Qubit ds DNA HS assay followed by normalization to 2 nanomoles using serial dilution where the highest concentrated library was diluted to 100 nmol > 40 nmol > 4 nmol and finally 2 nmol. DNA and cDNA libraries were pooled separately. Since this was a standardization study, libraries below 2 nmol were taken neat. Finally, DNA and RNA pooled libraries were mixed in a 7:3 ratio, respectively, followed by denaturation, and 9.5 pmol libraries were loaded onto a MiSeq V2 Kit spiked with a 10% phiX library.

### 2.4. Sequencing Parameters

The Illumina experimental manager was used to generate the template sheet. Libraries were paired-end (2 × 150) sequenced on a V2 flow cell on MiSeq (Illumina San Diego, CA, USA). The run was monitored with respect to specifications outlined by the manufacturer (Illumina). Parameters such as cluster densities, reads passing filter and an output greater than Q30 values were considered as the first quality indicator of the run. Data generated were analyzed using the in-house developed bioinformatics tool “ClinOme”. Quantitative Multiplex Reference Standard (FFPE) from Horizon was included in each batch of library preparation to ensure the quality of the run.

### 2.5. Statistical Analysis

A random selection of the samples was carried out. The statistical analysis was performed using IBM SPSS 25. The distribution pattern of data was analyzed using a Shapiro–Wilk test. Since the data were not normally distributed, a statistical comparison was performed using the Mann–Whitney U test, and correlation was analyzed using Spearman’s rho test. *p* < 0.05 and *p* < 0.01 were considered significant.

### 2.6. Receiver Operating Characteristic Curve (ROC) Analysis

To evaluate the capacity of each parameter of DNA and RNA for predicting the functionality of libraries for reporting, ROC analysis was performed using IBM SPSS 25. The area under the curve (AUC), specificity, and sensitivity were calculated.

## 3. Results

### 3.1. Quality and Quantity of Samples

#### 3.1.1. FFPE DNA: DIN, DNA Concentration and Library Concentration

**Figure 1:**
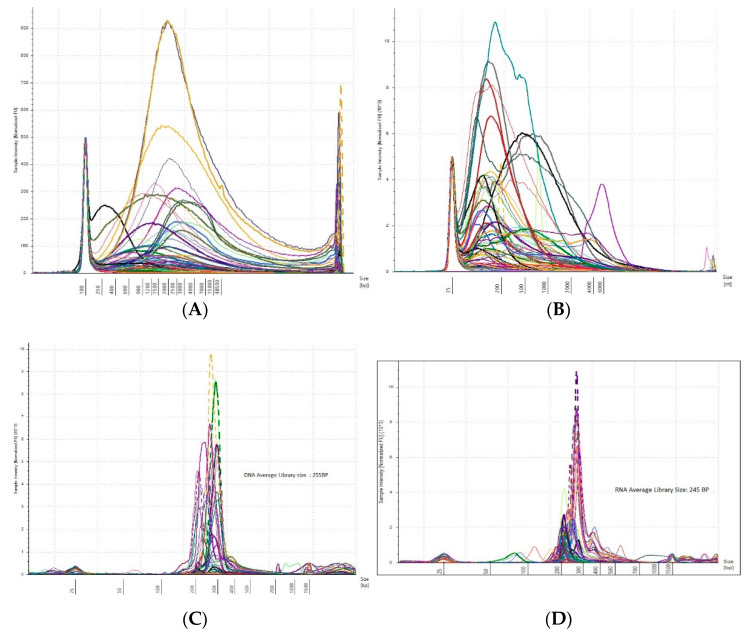
Quality check of FFPE DNA and RNA samples. (**A**,**B**): the fragment distribution of FFPE DNA and RNA samples on TapeStation, respectively. This shows the variation in nucleic acid extracted from FFPE samples before the library preparation (**Left** to **Right**). (**C**,**D**): the FFPE DNA library and RNA library, respectively. The average library size observed in the study for DNA is 255 bp and RNA is 245 bp (**Left** to **Right**).

**Figure 2:**
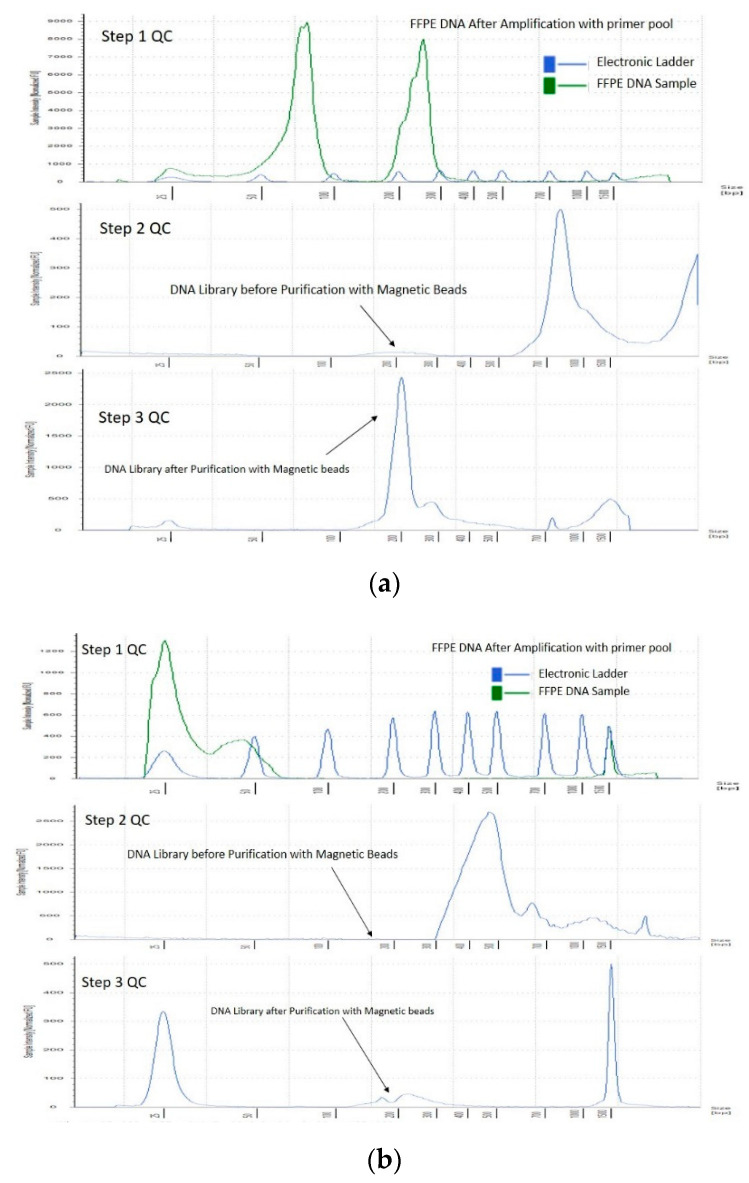
Three-step quality check of representative FFPE DNA samples. (**a**). Good on-target coverage DNA sample QC on TapeStation. (**b**) Poor on-target coverage FFPE DNA sample qc on TapeStation. Figure 2 shows a three-step quality check (QC) of representative FFPE DNA samples showing good and poor coverage on TapeStation, respectively. Step 1: QC after amplification with DNA primer pool, Step 2: QC after index adapter ligation to the amplified DNA sample, Step 3: Final library after purification with magnetic beads. The good coverage samples (**a**) had DIN: 5.9, DNA concentration: 110 ng/µL, library concentration: 170 nmol, average library size (bp): 230, coverage: 804×. The poor coverage sample (**b**) had DIN: 2.3, DNA concentration: 2.5 ng/µL, library concentration: 2.12 nmol, average library size (bp): 253, coverage: 3.5×.

A total of 144 DNA samples were divided into two groups based on on-target coverage and variant allele frequency (VAF) viz. ≤ 250× and VAF 10% and ≥250× and VAF 10%. Out of 144 FFPE DNA, 98 samples exhibited coverage above 250× and VAF 10% while 46 samples resulted in poor NGS quality, with coverage below 250× and VAF 10%. As suggested in the literature, DNA libraries with ≥250× coverage and VAF of 10% are considered good coverage samples while anything below this criteria was grouped into poor coverage samples [13]. The pre-library and post-library QC of FFPE DNA and RNA are shown in Figure 1. The three-step QC check provides the added advantage of monitoring the library preparation (Figure 2).

**Figure 3:**
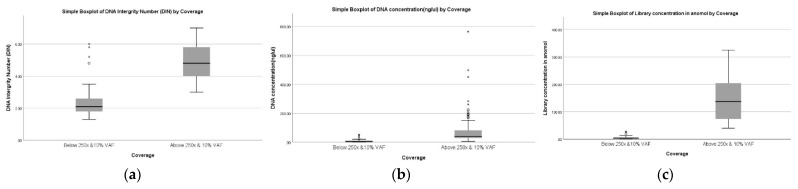
Graphical representation of parameters in FFPE DNA samples according to NGS coverage. (**a**): DIN, (**b**): DNA concentration, (**c**): DNA library concentration (**Left** to **right**). Figure 3 shows a box plot of FFPE DNA sample coverage with respect to parameters such as DIN, DNA concentration, and library concentration. All the parameters show a significant difference between the poor coverage (below 250× and 10% VAF) and good coverage (above 250× and 10% VAF). * represents the extreme data points, ◦ represents the ouliers data points.

The sample characteristics across two groups are displayed in Table 1 and a graphical representation is shown in Figure 3. Table 1 describes poor coverage samples (≤250× and VAF 10%) with a median DIN of 2.1 (1.3–6), initial DNA concentration of 4.99 ng/µL (2.39–53), and a library concentration of 3.955 nanomoles (0.37–27.65). Similarly, the good coverage samples (≥250×, VAF 10%) have a median DIN of 4.8 (3–7), an initial DNA concentration of 39 ng/µL (5.9–764) and a library concentration of 137.18 nanomoles (40.3–325). The Mann–Whitney U test indicated that quality (DIN) and quantity (DNA concentration and library concentration) were significantly higher in the good coverage sample than in the poor coverage sample (*p* < 0.05).

#### 3.1.2. FFPE RNA: RIN, DV100, DV200, RNA Concentration, Library Concentration

**Figure 4:**
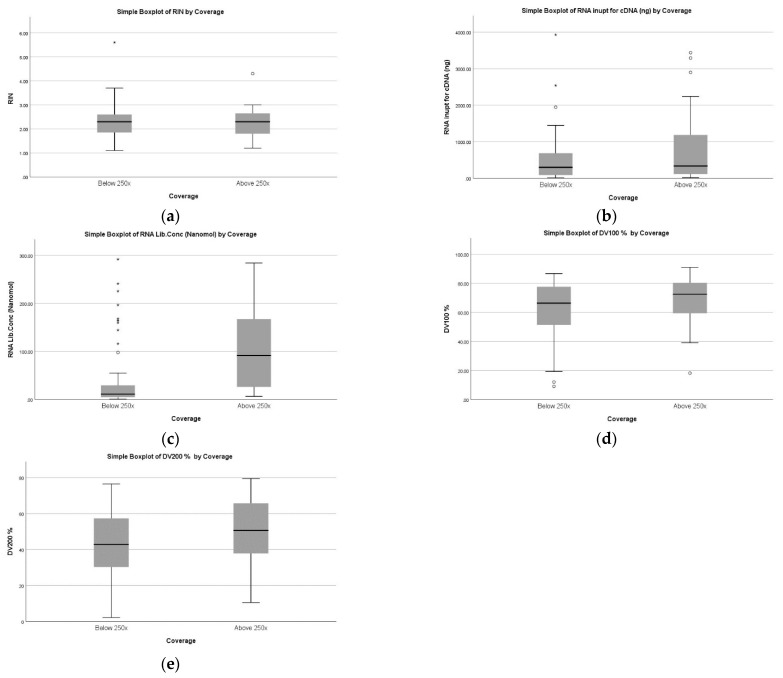
Graphical representation of FFPE RNA samples according to NGS coverage. (**a**): RIN, (**b**): RNA input for cDNA, (**c**): RNA library concentration, (**d**): DV 100, (**e**): DV 200 (**Left** to **Right**). The figure describes a box plot of FFPE RNA samples coverage with respect to parameters such as RIN, RNA input for cDNA, RNA library concentration, DV 100, and DV 200. Only RNA library concentration (nanomol) was found to be significantly different between poor coverage (below 250×) and good coverage (above 250×). * represents the extreme data points, ◦ represents the ouliers data points.

For better quality assurance of RNA along with different methods such as RIN number and DV 100 and DV 200, a PCR amplification assay of housekeeping genes (100 bp, 400 bp size) was performed. Table 2 of 103 RNA samples describes median values for the various parameters in group ≤250× and ≥250× with 5% VAF [14]. Out of 103, 39 samples were able to generate NGS results within the acceptable range (above 250× and 5% VAF) while 64 samples yielded poor quality of coverage which was below 250× and 5% VAF. In the group of ≤250×, the observed value for RIN was 2.3 (1.1–5.6), RNA concentration for cDNA was 299.96 ng (12.67–3927 ng), DV100 was 66.4% (9.15–86.77%), DV200 was 42.91% (2.24–76.44%), and library concentration was 11.14 nanomoles (0.71–292 nanomole). Similarly, the median values for the various parameters in the above 250× group are RIN of 2.3 (1.1–4.3), total RNA concentration of 327.6 ng (14.84–3437 ng) and library concentrations of 79.71 nmol (6.4–284 nanomol). However, only the library concentration value exhibited a statistically significant difference (*p* < 0.05) across both the groups (≤250× and ≥250× coverage) as per the Mann–Whitney U test (Figure 4).

### 3.2. Influence of Preanalytical and Post-Analytical Parameters on NGS Coverage

#### 3.2.1. FFPE DNA

Since there was a significant difference in quality and quantity between good and poor coverage samples, we must check the correlation between the overall NGS coverage with DIN, DNA concentration, and library concentration (nanomole). In a Spearman’s rho test (Table 3), it was observed that the overall coverage exhibited a strong positive correlation with DIN (rs = 0.703) and initial DNA concentration (rs = 0.717), while the library concentration showed a very strong positive correlation (rs = 0.808). All correlations showed significance at *p* < 0.01.

#### 3.2.2. FFPE RNA

Similar to the Spearman’s rho test of RNA samples, the library functionality was found to be correlated with only the RNA library concentration in nanomole (rs = 0.520, *p* < 0.01), while no correlation was found with other parameters (Table 3).

**Figure 5:**
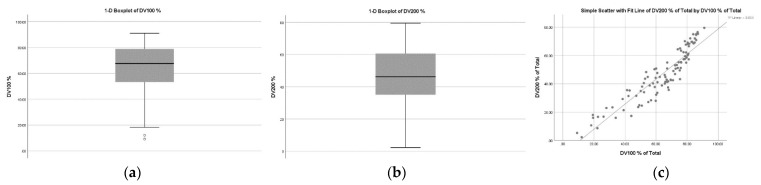
Graphical representation of overall distribution value (dv) in FFPE RNA samples. (**a**): DV100 (Overall) (**b**): DV 200 (Overall) (**c**): Correlation between DV100 and DV200 (**Left** to **Right**). (**a**,**b**) describe the box plot of DV 100 and DV200 for RNA samples in the study. (**c**): The correlation between DV100 and DV200 for RNA samples in the study. ◦ represents the ouliers data points.

However, it was also noticed that library concentration showed a weak correlation with DV200 (rs = 0.210, *p* < 0.05). The median value of DV100 was 63.62% and DV200 was 46.52% in FFPE RNA samples. These samples also exhibited a strong correlation between DV100 and DV200 (R^2^ = 0.854) (Figure 5). This suggests that compared to other parameters, the concentration of the library and DV 200 has the potential to generate functional libraries. DV 200 also showed an indirect correlation with library functionality. However, RIN, DV100, and total RNA concentration did not show a direct correlation with the library functionality in this study.

### 3.3. Receiver Operating Characteristic Curve Analyses

**Figure 6:**
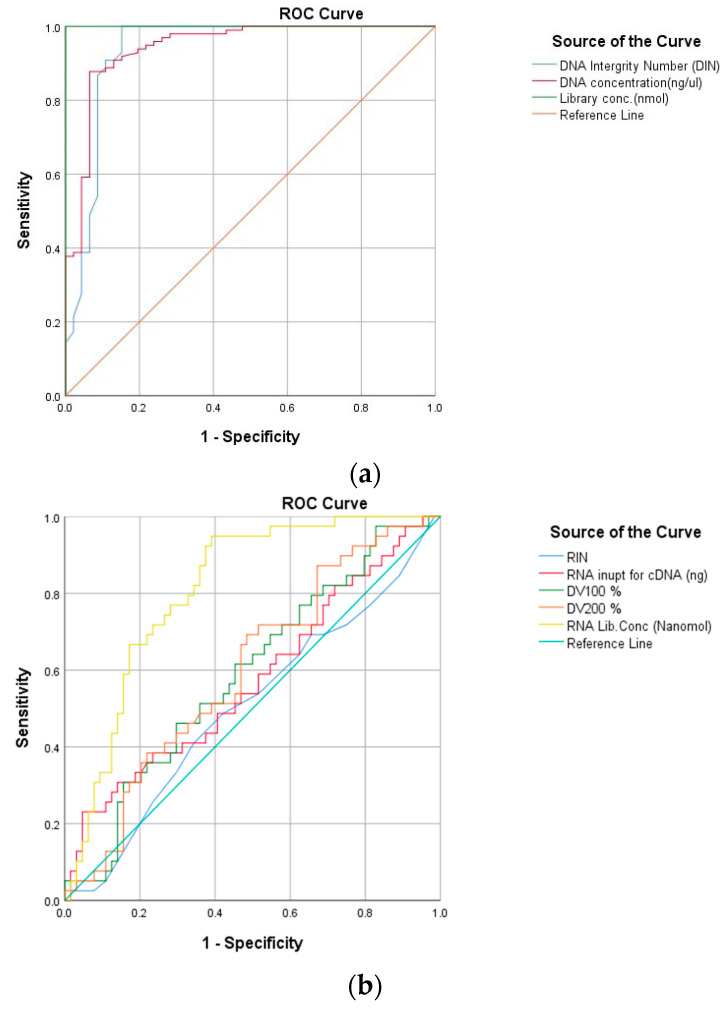
ROC analysis of FFPE samples. (**a**) DNA, (**b**) RNA.

This study intended to test the performance parameters of DNA and RNA responsible for producing good coverage libraries, hence ROC analysis was carried out (Figure 6). It was observed that DIN, DNA concentration, and DNA library concentration are good indicators, showing AUC 0.935, 0.944, and 1.00, respectively. In the case of RNA, the ROC curve indicated that RNA library concentration is a better indicator of the library functionality with AUC (0.810). However, RIN showed the least predictive value with AUC (0.504) followed by total RNA concentrations of AUC (0.574), DV100 (0.590), and DV200 AUC (0.598) (Table 4).

Figure 6a,b shows the receiver operating characteristic (ROC) curve analyses of pre-analytical and post-analytical parameters for DNA and RNA. For DNA, pre-analytical parameters are DIN and DNA concentration (ng/µL) while for RNA, RIN, RNA input for cDNA (ng), DV100, and DV200 are the pre-analytical parameters. The post-analytical parameter for DNA and RNA is library concentration (nanomole).

Based on ROC analysis, the parameters showing the highest area under curve (AUC) are considered for cut-off. (Table 5). These threshold values predicted the success of FFPE samples for a targeted NGS assay.

### 3.4. Decision Tree

**Figure 7:**
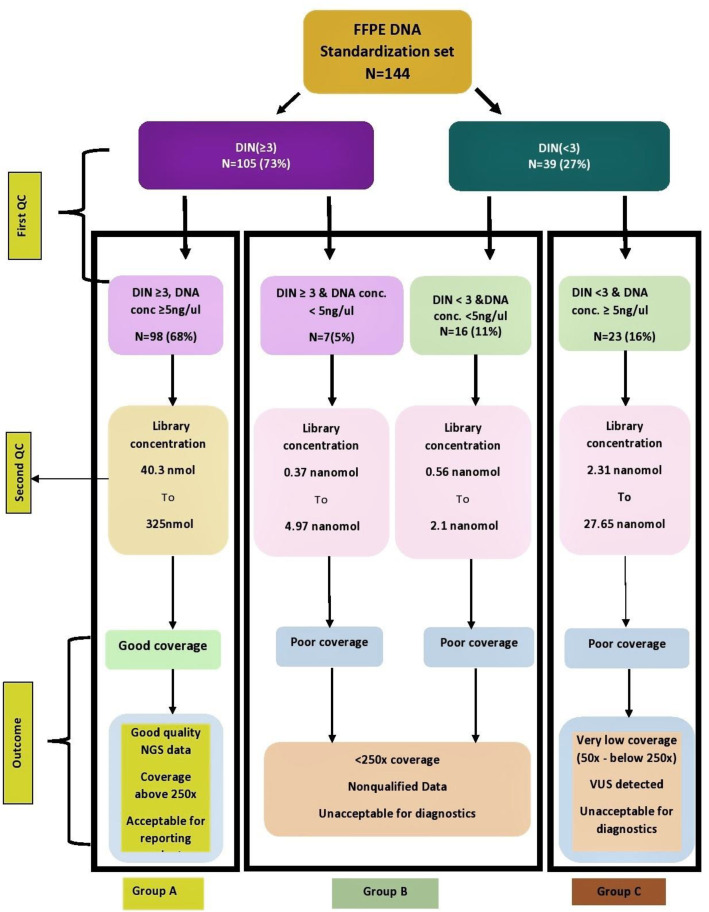
Decision tree for FFPE DNA. This figure describes the decision tree based on the pre-analytical and post-analytical parameters observed in 144 DNA samples. DIN, DNA concentration, and library concentration are found to be the predictive parameters while implementing targeted NGS in diagnostic settings. The first QC checkpoint is at the preanalytical phase consisting of DIN and DNA concentration. The second QC is at the library concentration of DNA samples. Implementing both QC checks can predict the outcome of FFPE DNA-based NGS for a targeted panel in a diagnostics setting.

Based on the overall study of FFPE samples and the statistical analysis, a decision tree for DNA was established. This provided better predictive values of parameters such as DIN, DNA concentration, and library concentration while implementing targeted NGS in diagnostic settings (Figure 7).

## 4. Discussion

In this study, it is observed that the quality and quantity of nucleic acid extracted from FFPE tissue are interdependent parameters that can affect the NGS library quality as well as data generated after sequencing. The study also suggests that FFPE samples with DIN < 3 as well as DNA concentration < 5 ng/µL are likely to fail (Figure 7, Group C). Although the Ampliseq protocol says DNA concentrations in the range 1–100 ng should be used, our study strongly suggests, for Indian FFPE blocks, which are substandard at times, not going below a 5 ng/µL DNA concentration, which deviates from the original protocol.

Samples that have a DIN < 3 but concentration ≥ 5 or vice versa (Figure 7, Group B) may generate bad quality data with lower sequencing coverage.

Our study illustrates that if FFPE DNA has a lower DIN value, i.e., it is highly fragmented where the concentration is greater than 5 ng/µL, such samples will also generate poor quality NGS data, therefore, compensating the poor DNA quality with higher DNA concentration is likely to give bad quality NGS data resulting in coverage less than 250× with variants of unknown significance.

This study suggests that there are fewer chances of library failures provided samples had a minimum DIN of 3 with a DNA concentration of at least 5 ng/µL and a minimum library concentration of 40 nmol (Figure 7, Group A).

As per the literature survey, limited studies are present where QC values such as DIN and DNA concentration are evaluated together for FFPE samples as preanalytical factors for amplicon-based NGS. The earlier studies were focused on pH-based ion torrent semiconductor detection technology, while our standardization uses the Illumina NGS platform where sequencing by synthesis is utilized with fluorescence-based detection systems [12].

Bonfiglio et al. [15] used a DIN cut-off of 3 for the whole exome capture system, while David Millan-Esteban [16] showed that a DIN of <2.05 and a quality control value of >5.63 were not suitable for amplicon-based NGS of melanoma samples in storage for more than 7 years. Although the cohort studied by David Millan-Esteban et al. comprised only 59 samples, our study agrees with the previous findings [12].

Overall, our study targeted two-year-old FFPE samples, but not for any specific cancer type. We have demonstrated that purely DIN (≥3), DNA concentration (≥5 ng/µL), and library concentration (≥40 nanomol) are the factors that were able to discriminate the poor coverage and good coverage libraries. A prior study on NGS of cytology samples describes that the success of NGS can be positively correlated with DNA yield [17], while our study suggests that both DNA integrity and optimal DNA concentration are the prime pre-analytical factors responsible for successful NGS libraries of FFPE tissue samples.

This study shows that the abovementioned pre-analytical considerations are the benchmark parameters suitable for predicting the success or failure of FFPE samples for amplicon-based libraries. FFPE samples rejected due to the stringent parameter for NGS assays may be considered for orthogonal testing methods such as real-time PCR, ddPCR, Sanger sequencing, etc. To validate the LOD of the targeted panels, specimens can be diluted to obtain various allele frequencies, followed by a different range of DNA concentrations [18]. In addition, DNA standards of different DIN are also commercially available.

The decision tree approach in the study can be set as a benchmark while working with FFPE samples for molecular testing. Our approach to the targeted panel gives accurate results and thus can become a good laboratory practice for NGS-based testing of FFPE samples in diagnostic laboratories.

Our PCR amplification assay of housekeeping genes has an added advantage for quality confirmation and selection of cDNA with amplifiable transcripts [19]. For FFPE RNA, we observed that RIN is not an accurate quality indicator. Although the RNA samples exhibited satisfactory DV scores in terms of DV100 and DV200, there was no significant difference in DV values with respect to library functionality. Similar results were observed in terms of RNA input concentration for cDNA. For successful RNA libraries, our data strongly suggest that RNA library concentration is an important predictive parameter. This indicates that setting threshold values for RNA quality remains a challenge for FFPE samples.

This study of standardization and optimization of NGS libraries was undertaken in order to minimize the time and the cost of NGS assays. There are also other factors involved in successful assays such as the number of genes and amplicons of the panel, various types of NGS platforms, the total number of samples loaded per batch, and the number of denatured libraries loaded on the flow cell. V2 and V3 sequencing chemistries also affect the coverage of NGS samples. Hence, we suggest that all the above parameters must be considered while standardizing the NGS assays.

Thus, the above benchmark parameters helped to overcome the failure of libraries, and acceptance of FFPE samples in the NGS clinical laboratory. In order to establish a good clinical laboratory practice, this study will be very useful in maintaining the turnaround time (TAT).

## 5. Conclusions

This study is possibly the first to report the significance of DIN, DNA concentration, and library concentration as predictive combined parameters for successful libraries with good coverage. We can set the following parameters for good coverage of the samples:

1. DNA integrity number (DIN), DNA concentration, and library concentration (nanomole) were associated with the coverage. These values showed significant differences related to sample coverages (above and below 250× with 10% VAF).

2. RNA library concentration is the only parameter directly associated with coverage. However, DV200 showed a correlation with RNA library concentration. The RNA distribution value (DV) value is a better quality metric than the RNA integrity number (RIN). The samples in this study showed a strong correlation between DV100 and DV200 (R^2^ = 0.854). They also did not show any statistically significant difference between DV100 and DV200 with respect to library functionality.

3. A possible reason might be the FFPE blocks were a maximum of 2 years old, which expressed the median value of DV100 as 63.62% and DV200 as 46.52%, which are within the acceptable range for NGS. However, the literature indicates that the old FFPE blocks of DV100 should be considered over DV200 [20].

4. The ROC curve classified the DIN, DNA concentration and library concentration of DNA and RNA as a performance factor to procure coverage above 250×.

5. Last but not least, we strongly recommend cDNA multiplex housekeeping gene PCR assays for quality assurance of the RNA samples.

Thus, this systematic study helped to establish thresholds that can generate a minimum coverage of 250× with a 10% VAF of the gene alteration, such as a minimum requirement of DIN 3, a DNA concentration of 5 ng/µL, a DNA library concentration of 40 nanomoles, and an RNA library concentration of 30 nanomoles.

## 6. Limitations and Future Recommendations

The percentage of tumor cells in FFPE specimens can affect the data output. The adjacent non-tumor tissue contaminates the tumor content of the FFPE specimen, thereby undermining the genomic studies. Macro dissection is the preferred method to augment the tumor content of the sample. Due to analytical limitations in this study, macro dissection was bypassed and a baseline of 20% tumor cellularity was set as the threshold value. Samples showing 250× coverage and 10% VAF have a high possibility of false-negative results and low allele frequency mutations; however, a coverage of 500× with 5% VAF is recommended in the FFPE samples. The quality and quantity of FFPE samples may differ depending on the sequencing chemistry, number of genes in the panel, target size, and number of sample pools per run. However, there is a need for an independent study to detect molecular alterations with low allelic fractions in FFPE samples. Therefore, to conclude, performing wet-lab parameters without QC standards for NGS may generate inconsistent and non-reproducible results. Hence, in order to reduce the wastage of expensive reagents and repetition of the assay that leads to an increase in the TAT, this study will be helpful for decision-making in diagnostic labs.

Our NGS study provides straightforward DNA and RNA QC assessment parameters for FFPE samples, and aims to identify the failure of the samples. However, setting up a threshold for FFPE RNA samples remains uncertain. We strongly recommend cDNA multiplex housekeeping gene PCR assays for quality assurance of RNA in addition to a TapeStation.

We strongly recommend that the laboratories perform comprehensive optimization of the commercial kits before implementing and incorporating them into the service of a patient. Thus, to summarize, for FFPE-based NGS samples, DNA integrity number (DIN), DNA concentration, and DNA and RNA library concentrations can be quality checkpoints for successfully targeted panel-based NGS results.

## Figures and Tables

**Table 1:** Sample characteristics of FFPE DNA according to NGS coverage.

FFPE DNA							
Parameters	≤250× and VAF 10% (Poor Coverage) (*n* = 46)			≥250× and VAF 10% (Good Coverage) (*n* = 98)			*p*-Value
	Minimum	Median	Maximum	Minimum	Median	Maximum	
DNA Integrity Number (DIN)	1.3	2.1	6	3	4.8	7	<0.05
Initial FFPE DNA concentration (ng/µL)	2.39	4.99	53	5.9	39.175	764	<0.05
Library concentration (nanomol)	0.37	3.955	27.65	40.3	137.18	325	<0.05

**Table 2:** Sample characteristics of FFPE RNA according to NGS coverage.

FFPE RNA							
Parameters	≤250× and VAF 5% (Poor Coverage) (*n* = 64)			≥250× and VAF 5% (Good Coverage) (*n* = 39)			*p*-Value
	Minimum	Median	Maximum	Minimum	Median	Maximum	
RIN	1.1	2.3	5.6	1.2	2.3	4.3	0.948
RNA input for cDNA	12.67	299.95	3927	14.84	327.6	3437	0.21
DV100	9.15	66.4	86.77	18.25	71.055	91.04	0.128
DV200	2.24	42.91	76.44	10.61	49.13	79.46	0.095
RNA Library Concentration Nanomol	0.716	11.14	292	6.4	79.715	284.09	<0.05

**Table 3:** Correlation of pre-analytical and post-analytical parameters with coverage status.

Parameters	Correlation Coefficient w.r.t NGS Coverage	*p*-Value
FFPE DNA		
DNA Integrity Number (DIN)	0.703 **	<0.01
FFPE DNA concentration (ng/µL)	0.717 **	<0.01
Library concentration (nanomole)	0.808 **	<0.01
FFPE RNA		
RIN	0.006	0.949
RNA input for cDNA (ng)	0.124	0.211
DV100	0.151	0.128
DV200	0.165	0.095
RNA Lib Conc Nanomole	0.520 **	<0.01

** = Correlation is significant at the 0.01 level. All correlations were analyzed using Spearman’s rho test. The DNA preanalytical parameters are DIN and DNA concentration (ng/µL) while for RNA, RIN, RNA input for cDNA (ng), DV100, and DV200 are preanalytical steps. The post-analytical parameter for DNA and RNA is library concentration (nanomole).

**Table 4:** Area under curve (AUC) of ROC analysis for DNA and RNA parameters.

Parameters	Area under Curve	Standard Error
DNA		
DNA Integrity Number (DIN)	0.935	0.029
DNA concentration (ng/µL)	0.944	0.022
DNA Library Concentration (nmol)	1.000	0.000
RNA		
RNA Integrity Number (RIN)	0.504	0.059
RNA input for cDNA (ng)	0.574	0.059
DV 100%	0.590	0.057
DV 200%	0.598	0.057
RNA library concentration (nmol)	0.810	0.042

Results represented are at a 95% confidence interval (CI).

**Table 5:** The cut-off value of pre-analytical and post-analytical parameters for FFPE DNA and RNA.

Parameters	Area under Curve (AUC)	Std. Error	Cutoff Value	Sensitivity	1-Specificity
DNA Integrity Number (DIN)	0.935	0.029	3.00	1	0.152
FFPE DNA concentration (ng/µL)	0.944	0.022	5.00	1	0.5
Library concentration (nmol) (DNA)	1.000	0.000	40.65	0.99	0.00
Library concentration (nanomole) (RNA)	0.814	0.042	30.80	0.718	0.234

Table 5 The pre-analytical parameters are DIN and FFPE DNA concentration. Post-analytical parameters consist of the library concentration (nanomole) of DNA and RNA.

## Data Availability

The data analyzed in this study is subject to the following licenses/restrictions: As per HIPPA regulations, the datasets for this manuscript are not publicly available. Requests to access the datasets should be directed to A.C., email: anu.c1112@gmail.com.
